# Assessment of Pediatric Outpatient Visits for Notifiable Infectious Diseases in a University Hospital in Beijing During COVID-19

**DOI:** 10.1001/jamanetworkopen.2020.19224

**Published:** 2020-08-24

**Authors:** Zujin Luo, Shunli Li, Nan Li, Yichong Li, Ying Zhang, Zhixin Cao, Yingmin Ma

**Affiliations:** 1 Department of Respiratory and Critical Care Medicine, Beijing Engineering Research Center of Respiratory and Critical Care Medicine, Beijing Institute of Respiratory Medicine, Beijing Chao-Yang Hospital, Capital Medical University, Beijing, China; 2 Department of Infection and Disease Control, Beijing Chao-Yang Hospital, Capital Medical University, Beijing, China; 3 Research Center of Clinical Epidemiology, Peking University Third Hospital, Beijing, China; 4 Department of Clinical Research and Epidemiology, Fuwai Hospital Chinese Academy of Medical Sciences, Shenzhen City, China

## Abstract

This cross-sectional study assesses changes in numbers of pediatric outpatient visits for notifiable infectious diseases acquired through droplet transmission, contact transmission, or both after implementation of coronavirus disease 2019 (COVID-19)–related public health measures during the COVID-19 outbreak in Beijing, China.

## Introduction

In response to coronavirus disease 2019 (COVID-19), strict public health measures were implemented, and medical resources were enhanced to isolate and treat infected patients.^1^ Yet little is known about whether other types of hospital visits have been affected,^2^ especially visits for infectious diseases. We explored changes in pediatric outpatient visits for Chinese notifiable infectious diseases acquired through droplet transmission, contact transmission, or both during Beijing’s COVID-19 outbreak.

## Methods

We performed a cross-sectional study at Beijing Chao-Yang Hospital Western Branch in Beijing that followed the Strengthening the Reporting of Observational Studies in Epidemiology (STROBE) reporting guideline. The study was approved by the ethics committee of Beijing Chao-Yang Hospital, which waived the requirement for informed consent.

Using the hospital’s electronic medical records and notifiable diseases surveillance system, we identified all pediatric outpatient visits during the COVID-19 outbreak (January 19–April 15, 2020) and a matched control period in the previous year (January 19–April 16, 2019) and collected demographic and diagnostic information on patients with notifiable infectious diseases. During the COVID-19 outbreak, all febrile patients underwent nucleic acid testing of nasal and pharyngeal swab specimens to rule out COVID-19. The primary outcomes were the number of pediatric outpatient visits, number of pediatric patients with notifiable infectious diseases, and proportion of pediatric patients with notifiable infectious diseases in pediatric outpatient visits. We compared continuous variables with the Mann-Whitney *U* test and median differences, coefficient intervals, and categorical variables with the χ^2^ test or Fisher exact test. Two-tailed *P* < .05 defined statistical significance. All analyses were performed using SPSS version 25.0 (IBM).

## Results

A total of 2420 pediatric outpatient visits (median patient age, 4 [interquartile range, 2-6] years; 1325 [55%] male) were identified during the COVID-19 outbreak, an average of 28 per day, compared with 14 557 and 165, respectively, in 2019 (ie, an 83% decrease; Figure, panel A). Thirty-four patients with notifiable infectious diseases were reported during the outbreak, an average of 0.4 per day, compared with 383 and 4.3, respectively, in 2019 (ie, a 91% decrease; Figure, panel B). The proportion of patients with notifiable infectious diseases in pediatric outpatient visits (difference, –1.2%; 95% CI, –1.7% to –0.6%; *P* < .001), especially the proportion with influenza (difference, –1.3%; 95% CI, –1.8% to –0.8%; *P* < .001), was significantly lower during the outbreak than in 2019.

**Figure.**
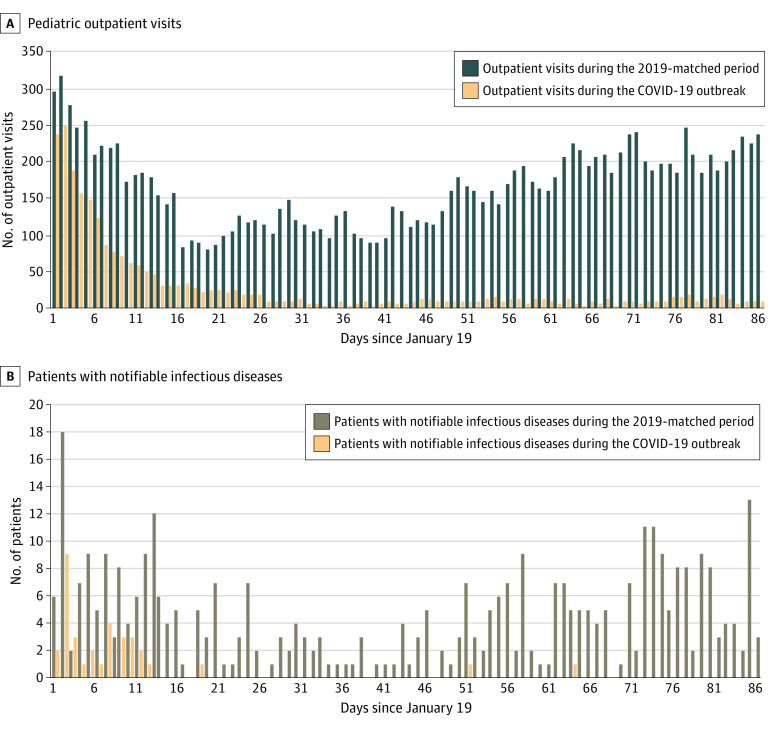
Daily Number of Pediatric Outpatient Visits and Patients With Notifiable Infectious Diseases, Beijing Chao-Yang Hospital Western Branch, in 88 Days Since January 19 in 2019 and 2020 The COVID-19 outbreak was from January 19 to April 15, 2020, and the 2019-matched period was from January 19 to April 16, 2019. COVID-19 indicates coronavirus disease 2019.

Demographic and diagnostic characteristics are summarized in the Table. The sex distribution was similar between the 2 periods (difference, –3.1%; 95% CI, –19.5% to 13.9%; *P* = .73), but the median age was younger during the outbreak (difference, –1 year; 95% CI, –2 to 0 years; *P* = .004). No pediatric patients with COVID-19 were confirmed in this hospital.

**Table.** Characteristics of Pediatric Outpatients With Notifiable Infectious Diseases During the COVID-19 Outbreak and a 2019-Matched Period^a^

	COVID-19 outbreak (n = 34)	2019-matched period (n = 383)	Difference (95% CI)^b^	*P* value
Sex, No. (%)				
Male	16 (47)	192 (50)	−3.1 (−19 to 14)	.73^c^
Female	18 (53)	191 (50)		
Age, median (IQR), y	3 (1 to 4)	4 (2 to 6)	−1 (−2 to 0)	.004^d^
Age distribution, y, No. (%)				
<1	3 (9)	13 (3)	5.4 (−1.6 to 21.5)^e^	.001^f^
≥1 to <5	26 (76)	181 (47)	29.2 (10.5 to 42.4)^e^	
≥5 to <10	4 (12)	153 (40)	−28.2 (−37.6 to 10.9)^e^	
≥10 to <14	1 (3)	36 (9)	−6.5 (−10.9 to 7.9)^e^	
Source, No. (%)				
Shijingshan district	27 (79)	286 (75)	4.7 (−13.6 to 16.9)^e^	.77^f^
Beijing other than Shijingshan	7 (21)	93 (24)	−3.7 (−15.9 to 14.6)^e^	
Outside Beijing	0 (0)	4 (1)	−1 (−2.8 to 11.6)^e^	
Daily confirmed patients with notifiable infectious diseases, median (IQR)	0 (0 to 0)	3.5 (2 to 7)	3.0 (3.0 to 4.0)	<.001^d^
Proportion of notifiable infectious patients in total outpatients, % (95% CI)^g^	1.4 (1.0 to 2.0)	2.6 (2.4 to 2.9)	−1.2 (−1.7 to −0.6)	<.001^c^
Notifiable infectious disease, No. (%)				
Influenza	27 (79)	358 (93)	−14.1 (−32.0 to −2.4)^e^^,^^h^	.02^f^
Hand, foot, and mouth disease	1 (3)	8 (2)	0.9 (−2.7 to 15.0)^e^	
Chickenpox	3 (9)	8 (2)	6.7 (−0.01 to 22.8)^e^	
Mumps	3 (9)	6 (2)	7.3 (0.5 to 23.3)^e^	
Scarlet fever	0	2 (1)	−0.5 (−2.1 to 12.1)^e^	
Acute hemorrhagic conjunctivitis	0	1 (0.3)	−0.3 (−1.7 to 12.4)^e^	

Abbreviations: COVID-19, coronavirus disease 2019; IQR, interquartile range.

^a^ The COVID-19 outbreak was from January 19 to April 15, 2020, and the 2019-matched period was from January 19 to April 16, 2019.

^b^ For categorical variables, the CI for the difference between two independent proportions was calculated with the Wilson approach. For continuous variables, the CI for the median difference was calculated with the Hodges-Lehmann approach.

^c^ By χ^2^ test.

^d^ By Mann-Whitney *U* test.

^e^ The 95% CI was calculated including continuity correction.

^f^ By Fisher exact test.

^g^ Proportion based on all outpatients (2420 for the COVID-19 outbreak period and 14 557 for the 2019-matched period).

^h^ Among patients with notifiable infectious disease, the decrease in the number of patients with influenza was most significant.

## Discussion

We found a decrease in pediatric outpatient visits for notifiable infectious diseases in a university hospital in Beijing during the COVID-19 outbreak. Except for scarlet fever (transmitted only via droplet) and acute hemorrhagic conjunctivitis (transmitted only via contact), the notifiable diseases studied, especially influenza, infect people via either droplet or contact transmission.^3^ Strict implementation of public health control measures in response to COVID-19 might have inhibited droplet and contact transmission of common infectious viruses.^4^ Guardian fear of contracting COVID-19 in the hospital may be a reason for the reduced number of outpatients.^5^ However, the reduced proportion of patients with notifiable infectious diseases confirms that these diseases were somewhat contained during the COVID-19 outbreak, especially given that febrile patients are required to seek urgent medical care.

Limitations of this study include the single-center design, the use of retrospective data, and the possibility that not all patients were identified. However, because the study took place in a traditional university hospital, the main findings might be representative of the actual number of pediatric patients with notifiable infectious diseases in Beijing. Also, these findings highlight the importance of public health measures for controlling infectious diseases.
